# Enigmatic Diphyllatea eukaryotes: culturing and targeted PacBio RS amplicon sequencing reveals a higher order taxonomic diversity and global distribution

**DOI:** 10.1186/s12862-018-1224-z

**Published:** 2018-07-18

**Authors:** Russell J. S. Orr, Sen Zhao, Dag Klaveness, Akinori Yabuki, Keiji Ikeda, Watanabe M. Makoto, Kamran Shalchian-Tabrizi

**Affiliations:** 1 Section for Genetics and Evolutionary Biology (EVOGENE), Department of Biosciences, University of Oslo, Kristine Bonnevies hus, Blindernveien 31, 0371 Oslo, Norway; 2 Centre for Integrative Microbial Evolution (CIME), Section for Genetics and Evolutionary Biology (EVOGENE), Department of Biosciences, University of Oslo, Kristine Bonnevies hus, Blindernveien 31, 0371 Oslo, Norway; 3 Department of Molecular Oncology, Institute of Cancer Research, Oslo University Hospital-Radiumhospitalet, Oslo, Norway; 4 Medical Faculty, Center for Cancer Biomedicine, University of Oslo University Hospital, Oslo, Norway; 5 Section for Aquatic Biology and Toxicology (AQUA), Department of Biosciences, University of Oslo, Oslo, Norway; 6 Japan Agency for Marine-Earth Science and Technology (JAMSTEC), 2-15 Natsushima, Yokosuka, Kanagawa 237-0061 Japan; 7 Faculty of Life and Environmental Sciences, University of Tsukuba, 1-1-1 Tennodai, Tsukuba, Ibaraki, 305-8572 Japan

**Keywords:** Diphyllatea, PacBio, rRNA, Phylogeny, Collodictyon, Amplicon

## Abstract

**Background:**

The class Diphyllatea belongs to a group of enigmatic unicellular eukaryotes that play a key role in reconstructing the morphological innovation and diversification of early eukaryotic evolution. Despite its evolutionary significance, very little is known about the phylogeny and species diversity of Diphyllatea. Only three species have described morphology, being taxonomically divided by flagella number, two or four, and cell size. Currently, one 18S rRNA Diphyllatea sequence is available, with environmental sequencing surveys reporting only a single partial sequence from a Diphyllatea-like organism. Accordingly, geographical distribution of Diphyllatea based on molecular data is limited, despite morphological data suggesting the class has a global distribution. We here present a first attempt to understand species distribution, diversity and higher order structure of Diphyllatea.

**Results:**

We cultured 11 new strains, characterised these morphologically and amplified their rRNA for a combined 18S–28S rRNA phylogeny. We sampled environmental DNA from multiple sites and designed new Diphyllatea-specific PCR primers for long-read PacBio RSII technology. Near full-length 18S rRNA sequences from environmental DNA, in addition to supplementary Diphyllatea sequence data mined from public databases, resolved the phylogeny into three deeply branching and distinct clades (Diphy I – III). Of these, the Diphy III clade is entirely novel, and in congruence with Diphy II, composed of species morphologically consistent with the earlier described *Collodictyon triciliatum*. The phylogenetic split between the Diphy I and Diphy II + III clades corresponds with a morphological division of Diphyllatea into bi- and quadriflagellate cell forms.

**Conclusions:**

This altered flagella composition must have occurred early in the diversification of Diphyllatea and may represent one of the earliest known morphological transitions among eukaryotes. Further, the substantial increase in molecular data presented here confirms Diphyllatea has a global distribution, seemingly restricted to freshwater habitats. Altogether, the results reveal the advantage of combining a group-specific PCR approach and long-read high-throughput amplicon sequencing in surveying enigmatic eukaryote lineages. Lastly, our study shows the capacity of PacBio RS when targeting a protist class for increasing phylogenetic resolution.

**Electronic supplementary material:**

The online version of this article (10.1186/s12862-018-1224-z) contains supplementary material, which is available to authorized users.

## Background

Diphyllatea is a protist group that holds a deep and distinct position in the eukaryote tree; dependent on the position of the root, it may represent one of the earliest diverging eukaryotic lineages [1–5]. Currently, only a few species are described using traditional microscopic methods. Initially, the class Diphyllatea and the order Diphylleida were proposed to encompass the biflagellate *Diphylleia rotans* and the quadriflagellate *Collodictyon triciliatum* [6]. However, revisions of the systematic classification changed this class to include the species *Sulcomonas lacustris* and several older synonyms (e.g. *C. sparseovacuolatum* for *C. triciliatum* and *Aulacomonas submarina* for *D. rotans*) [7, 8]. Currently, based on morphological features, with only 18S rRNA provided from *D. rotans*, Diphyllatea is proposed to consist of the three genera *Collodictyon*, *Diphylleia* (not to be confused with the homonym in Botanical nomenclature) and *Sulcomonas* [9, 10], with the first two constituting the order Diphylleida and the third family Sulcomonadidae. The three-representative species (*C. triciliatum*, *D. rotans* and *S. lacustris*) have been previously investigated by light and electron microscopy [8, 11–16]. They share a heart- or egg-shape form and possess a ventral groove [12, 14, 17], more or less dividing the body longitudinally. The size range of the identified species is variable (15–60 μm length for *Collodictyon,* 20–25 μm length for *Diphylleia* and 8–20 μm length for *Sulcomonas*).

As the description of species diversity and the erection of the whole taxonomic unit of Diphyllatea were based on microscopic observations, one could expect that sequencing surveys of environmental DNA would detect a larger number of cryptic species. On the contrary, excluding the original *D. rotans* 18S rRNA (AF420478) from Brugerolle et al. 2002, only a single partial 18S rRNA sequence of Diphyllatea has been reported from a Tibetan freshwater lake (AM709512), and until now, no Diphyllatea have been classified from other water samples [18]. It is known that environmental PCR using group-specific primers can effectively amplify the diversity of some unicellular eukaryotes [19–21], but such an approach has never before been applied to Diphyllatea. Hence, its diversity may currently be underestimated. Accordingly, the know geographical distribution of the class, based on molecular data, is currently limited to China, France and Norway, with less geographic resolution at lower taxonomical levels [1, 8, 18]. Though, morphological data seemingly suggests a possible global distribution of *C. triciliatum* [11, 14, 22–24].

Thus, the objective of this study is to investigate possible cryptic diversity and distribution of Diphyllatea by firstly studying the morphology of novel cultured strains, and secondly by the amplification and phylogenetic inference of Diphyllatea rRNA from marine and freshwater samples. Additional database mining will allow for the confirmation of the classes diversity and distribution.

To achieve a robustly resolved phylogeny that will allow us to infer the relationships within Diphyllatea, long rRNA sequence reads are essential. For this reason, we targeted Diphyllatea environmental DNA and sequence near full-length 18S rRNA gene amplicons with PacBio RSII technology.

Recently, eukaryotic diversity studies have used Illumina (predominantly MiSeq) technology for sequencing rRNA amplicons. As Illumina has a restricted read length, diversity studies have been limited to an amplicon maximum of approximately 450 bp. A result of this being that studies have either focused on short hypervariable regions of 18S rRNA [25–27], ITS [28, 29], or 28s rRNA [30, 31]. These regions, despite being variable, sometimes lack enough sequence variation to be able to divide some genera to the species level. Further, focusing on separate, non-overlapping rRNA regions makes it difficult to study amplicons in a comparative phylogenetic context. Conversely, the study of long amplicons has traditionally involved cloning and Sanger sequencing [32–34], a time-consuming and costly method when high depth is desired. The Pacific Bioscience (PacBio) RS sequencing platform offers an alternative to short Illumina reads by providing long (> 20 kb) sequencing reads. PacBio RS also represents an alternative to cloning and Sanger sequencing for longer rRNA amplicons. To date, PacBio RS has mainly been applied to genome and more recently transcriptome sequencing [35–38]. However, a few studies have shown the platforms viability for studying 16s rRNA diversity of prokaryotes [39, 40], and more recently eukaryotic rRNA amplicons [41, 42]. Though at present, no studies have applied PacBio to sequence targeted 18S rRNA amplicons of lengths > 1000 bp.

## Methods

### Culture isolation and maintenance

Asian strains of Diphyllatea were established by a single-cell isolation method from localities in Japan, Thailand and Vietnam (Table 1). The isolated strains from Asia were inoculated into the freshwater medium URO [43] with endogenous cyanobacteria (*Microcystis*, strain no. NIES-44) as food and established as cultures. The investigated Norwegian strain of *Collodictyon triciliatum* (i.e. strain Å85) was a clonal isolate (from a single-cell) from Lake Årungen initially cultured on WC-medium [44] with the cryptomonad *Plagioselmis nannoplanktica* or a strain of the green alga *Chlorella* as food [14]. Subsequently, all cultures were kept in BG11 ½ medium [45], with *Microcystis* strain CYA-43 provided by the Norwegian Institute of Water Research (NIVA–www.niva.no). All cultures were grown using the following conditions: 17 °C, 250 μMol m^− 1^ s^− 1^ of daylight-type fluorescent light at a 14/10 (L/D) cycle.

**Table 1:** Sampling locations for cultured Diphyllatea-like organisms and environmental DNA

Strain/sample	Sampling locality
Cultured samples	
Å85	Lake Årungen, Ås, Norway (59°41’N 10°44′E)
KIINB	Lake Inba, Thiba, Japan (35°44’N 140°10′E)
KIKNR01, 02, 03	Kaen Nakon Reservoir, Thailand (16°24’N 102°50′E)
KIVT01, 02, 03, 04	Hồ Dầu Tiếng, Vietnam (11°23’N 106°17′E)
KIVTT01, 02	Turtle Farm, Ha Tinh, Vietnam (18°19’N 105°53′E)
Freshwater DNA	
Årungen	Lake Årungen, Ås, Norway (59°41’N 10°44′E)
BOR41	Lake by Kinabatangan river, Borneo, Malaysia (5°25’N 117°56′E)
BOR42	Pond A, Sandakan, Borneo, Malaysia (5°50’N 118°7′E)
BOR43	Pond B, Sandakan, Borneo, Malaysia (5°50’N 118°7′E)
LD_DERW20	Derwent water, UK (54°34’N 3°8’W)
LD_ESTH20	Esthwaite water, UK (54°21’N 2°59’W)
LD_BASS2, 20	Bassenthwaite lake, UK (54°40’N 3°13’W)
SA78, 81	Pond, Cape Town, South Africa (33°56’S 18°24′E)
Marine DNA	
NB038	Naples Bay, Italy (40°49’N 14°18′E)
RA119	Roscoff, France (48°43’N 4°2’W)
VA105	Varna, Bulgaria (43°11’N 28°0′E)
20F268	Oslo Fjord, Norway (59°27’N 10°32′E)

**Fig. 1:**
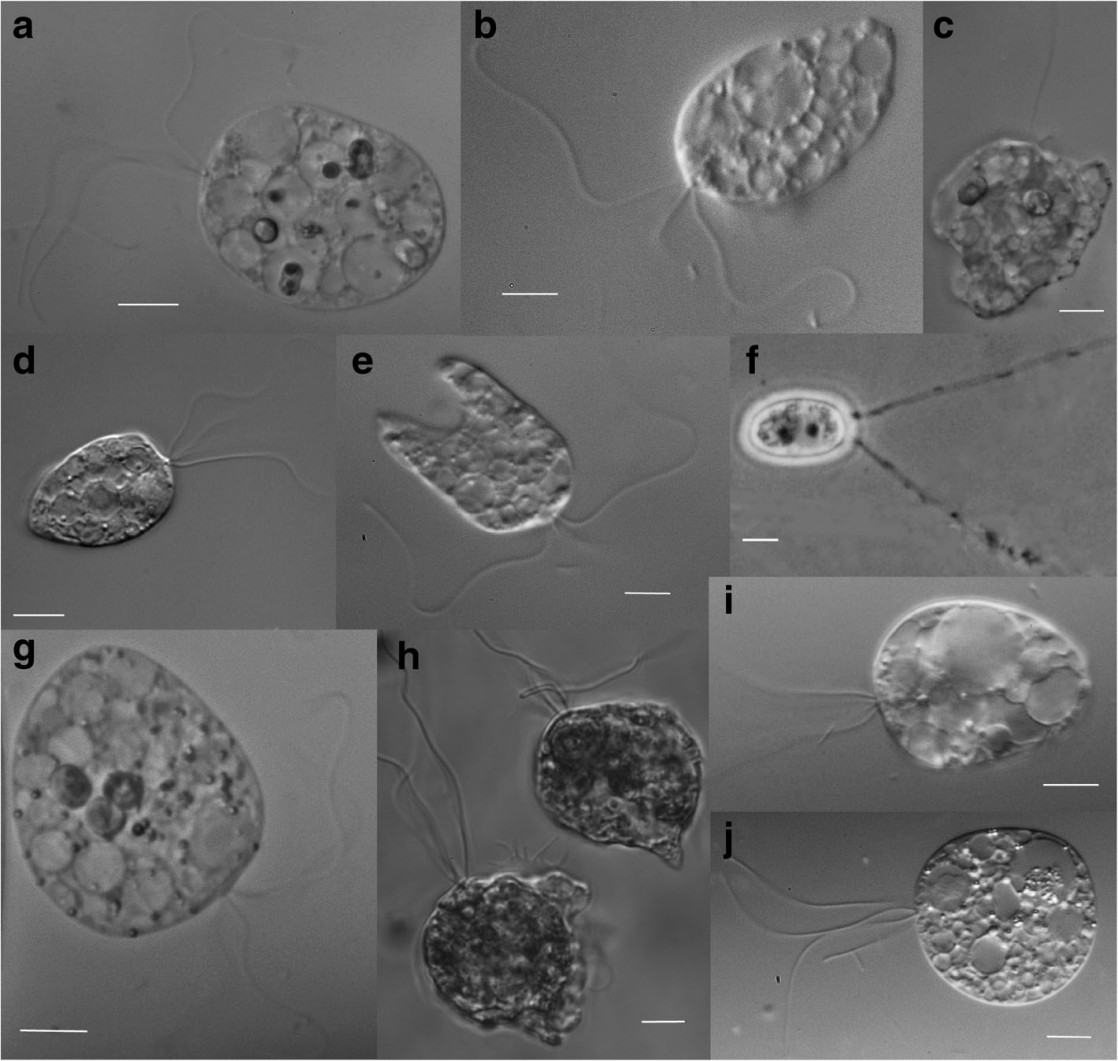
DIC Micrographs of newly cultured Diphyllatea-like organisms (Asian strains) and *C. triciliatum* (strain Å85). **a** KIVTT01. **b** KIINB. **c** KIKNR01. **d** KIVTT02. **e** KIKNR02. **f** Å85. **g** KIVT04. **h** Å85. **i** Å85. **j** KIVT01; scale bar 10 μm. Diphy II is represented in (**h** & **i**), whilst Diphy III is represented in (**a-e**), (**g** & **j**). Swimming cells are represented in (**b**, **d**, **e**, **i** and **j**). Amoeboid property represented in (**c** and **h**). Cell digestion shown in (**a** and **g**). Ventral furrow or groove depicted in (**d** and **i**). The resting stage or cyst is shown in (**f**) and is a phase contrast image

Environmental DNA samples were chosen based on size fractions encompassing the known cell size of Diphyllatea species/strains: 8–60μm (see Fig. 1, [6] and [8]). All environmental DNA was sampled subsurface

### Microscopy

Light microscopy of the 11 Diphyllatea was conducted using a Nikon Diaphot inverted microscope. Differential interference contrast (DIC) micrographs and video of Diphyllatea cells were taken using a Nikon D- series digital camera (D1 and D300S) connected to a screen. Electron microscopy (EM) was done by the negative staining of whole cells, after drop fixation on grids by osmium vapour [14].

### DNA isolation, PCR and sequencing

DNA was isolated from 50 ml of each culture by pelleting cells by centrifugation at 500 x g and 4 °C for 5 minutes, followed by standard CTAB chloroform/isoamylalcohol extraction and subsequent ethanol precipitation [46]. A ~6.3 kb region of the rRNA operon, covering the 18S, ITS1, 5.8S, ITS2 and 28S regions, was amplified as one continuous fragment with the forward primer NSF83 and the reverse primer LR11 (Table 2) utilizing Phusion High-Fidelity DNA polymerase, 35 cycles and a 50 °C annealing (ThermoFisher). The single ~6.3 kb PCR products were cleaned using Chargeswitch PCR Clean-up kit (ThermoFisher) and then Sanger sequenced (GATC Biotech, Germany) as separate fragments, utilizing primers outlined in Additional file 1: Table S2. Additional sequencing primers were designed using Primaclade [47]. The separate fragments were subsequently quality checked and assembled using the Phred/Phrap/Consed package [48] under default settings. Additional manual editing of the 11 contigs was performed in Mesquite v3.1 [49].

**Table 2:** List of primers used in this study

Primer name	Primer direction	Primer sequence (5′-3′)	*Tm* (°C)	Annealing site (5′-3′)	Reference or source
NSF83	F	GAAACTGCGAATGGCTCATT	49.7	84–103	[73]
Diphy257F	F	AAGWGGARTCATAATAACTTTTGCG	51.1	257–281	This study
Diphy453F	F	CGCAAATTACCCAATCCTG	48.9	453–471	This study
Diphy1881R	R	CGACCAAAACTCCAAAGATTTC	51.1	1860–1881	This study
1528R	R	TGATCCTTCTGCAGGTTCACCTAC	57.4	2127–2150	Adapted from [74]
SR1	R	CGGTACTTGTTCGCTATC	48	3565–3583	Ema Chao pers. comm
LR11	R	GCCAGTTATCCCTGTGGTAA	51.8	6414–6433	[75]

Primer annealing site is based on *Collodictyon* KIVT02 sequence, start is 83 bp prior to account for NSF83s annealing site. *Tm* is calculated using oligocalc [52]. Primers used for Sanger sequencing are listed in Additional file 1: Table S1. The 18S rRNA gene primers Diphy257F and Diphy1881R are Diphyllatea-specific

### Primer design and specificity confirmation

For optimal primer design with high specificity to the Diphyllatea clade, all available orthologous Diphyllatea sequences were used for alignment construction; Å85 and KIVT02 rRNA were used as blastn queries to extract Diphyllatea sequences deposited in the NCBInr database using default parameters. Further, *Diphylleia rotans* NIES-3764 rRNA (isolated in Amakubo, Ibaraki prefecture, Japan), taken from an unconnected genome project, was additionally used as a query and included in subsequent analyses. The resulting sequences were aligned together with the 11 culture sequences using the MAFFT Q-INS-i method [50], considering secondary RNA structure (default parameters used). The alignment was then manually checked and edited using Mesquite v3.1 [49] before designing primers with Primaclade [47]. All potential primers were tested for specificity to the Diphyllatea clade by checking sequence identity against non-Diphyllatea sequences in the Silva rRNA database in addition to an rRNA alignment with a broad sample of eukaryotic taxa [51]. OligoCalc [52] was applied to check self-complementarity and calculate primer *Tm*. The Diphyllatea-specific primers with highest potential were utilized in PCRs to confirm amplification of Diphyllatea rRNA, with optimal annealing temperature being established, and the non-amplification of DNA template external to Diphyllatea (a DNA mix of 30 cultures held in our lab). Diphyllatea-specific rRNA 18S gene primers designed in this study are listed in Table 2.

### Environmental DNA and confirmation of Diphyllatea

Environmental DNA was sampled from Lake Årungen by collecting and filtering two liters of surface water through a Whatman GF/C glass-fiber filter with an effective pore size of 1.2 μm prior to DNA isolation. Dr. David Bass (NHM) kindly provided supporting freshwater DNA samples (Table 1) from Borneo, South Africa, and the UK. Dr. Bente Edvardsen (UoO) in collaboration with BioMarKs [53], kindly provided marine DNA samples (Table 1) from Bulgaria, France, Italy, and Norway. Eukaryotic DNA was confirmed for all samples by PCR with a 55 °C annealing temperature, using the universal 18S rRNA primers NSF83 and 1528R (Table 2). Diphyllatea clade-specific PCR was subsequently performed on all environmental DNA samples targeting the 18S rRNA gene region with the primers Diphy257F and Diphy1881R (~1624 bp: see Table 2) with a 55 °C annealing temperature. Additionally, the annealing temperature for the Diphyllatea clade-specific PCR (Diphy257F - Diphy1881R) was lowered by 5 °C to allow primers to anneal to possible novel Diphyllatea template rRNA with lower sequence identity. Finally, for those environmental templates that gave no PCR product with the above primer pair, a pair with a lower specificity to the Diphyllatea clade was employed; Diphy453F and 1528R (~1697 bp: see Table 2), with a 55 °C annealing, amplifies a range of eukaryotes including Diphyllatea. A positive (Å85 and KIVT02 DNA) and negative control were employed for all PCRs. Positive amplicons were cleaned using Chargeswitch PCR Clean-up kit (ThermoFisher) or the Wizard SV gel and PCR clean-up system (Promega) and used for down-stream processing.

### PacBio barcodes, library prep and amplicon sequencing

As a more economical and efficient alternative to cloning, PacBio RS II was employed to achieve higher sequencing depth of the long environmental rRNA amplicons. PCR primers with symmetric (reverse complement) PacBio barcodes (21 bp) were attached to the separate 18S rRNA gene amplicons by PCR: a 2μl 1:10 dilution of template DNA (18S rRNA gene amplicon) was used as input in a two-step PCR protocol using Phusion High-Fidelity DNA polymerase (ThermoFisher), with a 72 °C 90 s annealing and 20 cycles. The resulting PCR product was cleaned before successful attachment of PacBio barcodes was confirmed using Bioanalyzer (Agilent Technologies). A single SMRTcell was prepared and sequenced, multiplexing both the Diphy257F - Diphy1881R (~1624 bp) and the Diphy453F - 1528R (~1697 bp) amplicons (Table 3 and Additional file 1: Table S2). The Norwegian Sequencing Centre (NSC), Oslo, Norway, performed library preparation and sequencing. The Library was prepared using Pacific Biosciences 2 kb library preparation protocol, before sequencing with the PacBio RS II using P4-C2 chemistry. Filtering was performed using Reads of Insert protocol on the SMRT portal (SMRTAnalysis v2.2.0.p1 build 134,282). Default settings (Minimum number of passes = 1 and Minimum Predicted Accuracy = 0.9) were used.

**Table 3:** PCR and sequencing results for environmental 18S rRNA gene amplicons

Strain/sample	NSF83-1528R	Diphy257F-Diphy1881R	Diphy453F-1528R	PacBio sequences CCS = 1	OTUs	Diphyllatea OTUs
Freshwater DNA						
Årungen	Y	Y	–	16	4	4 (2, 14)
BOR41	Y	Y	–	396	43	6 (2, 40)
BOR42	Y	Y	–	20	4	4 (1, 17)
BOR43	Y	Y	–	525	9	3 (1, 12)
LD_DERW20	Y	N	N	–	–	–
LD_ESTH20	Y	N	Y	16	8	–
LD_BASS2, 20	Y	N	Y	14	10	–
SA78, 81	Y	N	Y	9	7	–
Marine DNA						
NB038	Y	N	Y	248	115	–
RA119	Y	N	Y	232	54	–
VA105	Y	N	N	–	–	–
2OF268	Y	N	Y	54	23	–

Y = PCR product, N = no PCR product. The value for the PacBio reads (column five) is minus chimeras identified in Uchime. For Diphyllatea OTUs (last column) the number in brackets represents firstly the number of OTUs constituting > 1 read, and secondly the total number of reads these non-unique OTUs constitute; these are represented in the rRNA phylogeny (Fig. 3 and Additional file 1: Figure S2). For additional sequencing results per PacBio barcode see Additional file 1: Table S2. The total diversity of all 281 OTUs generated in this study is provided in Additional file 1: Figure S4. Though briefly, and outside the scope of this study, for the marine samples (192 OTUs): bivalve, ciliate, diatom, dinoflagellate and segmented worm rRNA were most abundant. For the freshwater samples (25 OTUs): ciliate, cryptomonad and diatom rRNA were most common

### Database-mining for marine Diphyllatea

To further investigate any cryptic presence of Diphyllatea in marine environments, publicly available databases were mined. The BioMarKs, Global Ocean Sampling (GOS), Tara oceans marine metagenome, and Tara oceans V9 databases were all queried for presence of Diphyllatea rRNA using the *Diphylleia*, KIVT02 and Å85 *Collodictyon* rRNA sequences. Further, the GOS and Tara oceans marine metagenome databases were queried with the 124 *Collodictyon* gene transcripts previously used to infer the phylogenetic placement of Diphyllatea [1]. It should be noted that the known *C. triciliatum* transcriptome reported in Zhao et al. 2012, was generated from the same Å85 strain represented in this study [1].

### Clustering, alignment construction and phylogenetic analyses

PacBio sequencing reads were split into their respective samples and barcodes removed using the SMRT portal. Reads were subsequently filtered keeping a CCS read accuracy of 1.0 (high quality sequences constituting multiple read passes). CCS Reads lacking either the forward or reverse primer sequence were discarded. Reads were then clustered with a 98% identity threshold, utilizing the “-cluster-otus” command in Usearch v8.1 [54, 55]. The “-cluster-otus” command additionally removed possible chimeric reads from the dataset. Uncultured clones from the same locality, previously categorised as Diphyllatea, acquired from NCBInr, were also clustered at the same identity. Operational taxonomic unit (OTU) clustering was carried out in this study using a 98% clustering identity threshold that is more stringent than the more commonly used 97% threshold. OTU clustering at 97% has recently been shown to be too conservative for estimating diversity in microbial eukaryotes, merging different species in the same cluster [56, 57]. Further, a 97% clustering identity is usually applied to illumina amplicons when targeting variable rRNA regions [58], with a more stringent threshold being traditionally applied to longer read rRNA amplicons encompassing conserved and variable regions [56, 59]. Clusters (OTUs) were then queried with blastn against a private database on CLC main workbench 7 (Qiagen) containing a broad selection of eukaryotic 18S rRNA taxa, including Diphyllatea (Å85, *Diphylleia rotans* and KIVT02). OTU hits with an E-value of 0.0 to Diphyllatea were subsequently aligned to the previously constructed alignment using MAFFT “—add” with default parameters and manually refined with Mesquite v3.1 [49]. Phylogenetic placement of the OTUs was checked using RAxML (method described below), with only those clustering with the Diphyllatea clade and constituting > 1 read being kept for further analysis. Further, to check if a possible cryptic diversity was disregarded during filtering, the method was repeated on reads with a CCS accuracy < 1.0. After the removal of ambiguously aligned characters, using Gblocks with least stringent parameters [60], the final dataset consisted of 64 taxa and 3983 characters. The alignment (both masked and unmasked) has been made freely available through the authors’ ResearchGate pages (https://www.researchgate.net/home).

Maximum likelihood phylogenetic analyses were carried out using the GAMMA-GTR model in RAxML v8.0.26 [61]. The topology with the highest likelihood score of 100 heuristic searches was chosen. Bootstrap values were calculated from 500 pseudo-replicates. Bayesian inferences were performed using MrBayes v3.2.2 [62], applying the GTR + GAMMA+Covarion model. Two independent runs, each with three cold and one heated Markov Chain Monte Carlo (MCMC) chains, were started from a random starting tree. The MCMC chains lasted for 40,000,000 generations with the tree sampled every 1000 generations. The posterior probabilities and mean marginal likelihood values of the sampled trees were calculated after the burn-in phase, which was determined from the marginal likelihood scores of the initially sampled trees. The average split frequencies of the two runs were < 0.01, indicating the convergence of the MCMC chains.

To investigate any possible topological effect of inferring taxa with missing sequence data, a secondary alignment constituting the 18S rRNA was constructed (64 taxa and 1575 characters). This was inferred, as previous, and the topological congruence of the ingroup taxa with that of the larger rRNA dataset was tested using the Icong index (http://max2.ese.u-psud.fr/icong/index.help.html) [63]. Topologies were more congruent than expected by chance (Icong = 2.69 & *P*-value = 6.93e-12), rejecting any negative effect of inferring taxa with missing sequence data. As such, only the result for the larger rRNA analysis is presented (Fig. 3), with the 18S rRNA tree supplied as Supporting material (Additional file 1: Figure S2).

## Results

### Cultured Diphyllatea show a *Collodictyon* morphology

Our survey of the diversity of Diphyllatea generated 10 new strains from Japan, Thailand and Vietnam, which complemented the Norwegian *Collodictyon* strain already established (Table 1). Light microscope observations of all isolates showed a congruent morphology to *Collodictyon* by sharing an egg- or heart-like body and four isomorphic flagella (Fig. 1). All cultured strains were compared with the diagnoses of currently recognised species of Diphyllatea and assigned to the morphospecies *C. triciliatum* [11]. The swimming cells have a slow and relaxed movement, while rotating, driven by flagella (Fig. 1b, d, e, i, j and Additional file 2: Video S1). They have the ability to cling to the surface of the culture dish by a cytoplasmic veil and pseudopodia (i.e. an amoeboid property) within or from the sulcus (Fig. 1c, h and Additional file 3: Video S2). Observations of live cells show the central cytoplasm contains a few large or small vacuoles. Some of the vesicles contain food particles (i.e. *Microcystis* strain CYA 43) at various stages of digestion (Fig. 1a and g). All studied strains can form a ventral furrow or groove that extends dorsally, dividing the cell into two parts (Fig. 1d and i), but this is a non-permanent structure during the cell cycle. Emergence of a long groove may be exclusive to cells that are starting or initiating cell division. Furthermore, we uncovered thick-walled resting stages or cysts, with two long gelatinous filaments (Fig. 1f), similar to earlier descriptions of *Collodictyon* resting spores [64]. The resting stage was only observed in the Å85 culture, with more microscopy work needed to reveal if this feature is common among Diphyllatea species.

**Fig. 2:**
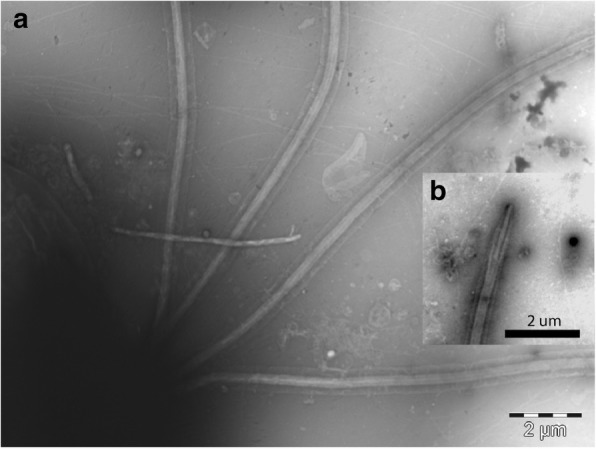
Electron micrographs of the flagella of *C. triciliatum* (strain Å85). **a** Four flagella and the membranes of flagella; **b** tip of flagellum

Electron microscopy of the negatively stained *Collodictyon* cells showed identical and smooth flagella lacking hairs or tomentum (Fig. 2), and that the periplast is hyaline and even. Other sub-cellular ultrastructures were difficult to identify; the cell is highly fragile and easily disrupted in the electron microscopy fixation process [14].

### Diphyllatea is divided into three higher order groups (Diphy I – III)

The rRNA fragments of ~6.3 kb for all 11 *Collodictyon* cultures were successfully amplified, sequenced and assembled (Accessions MF039356-MF039367).

With the inclusion of *Diphylleia rotans* rRNA (MF039365) the Diphyllatea class was divided into three ribotype groupings based on sequence length and indels; the amplified fragment ranged from ~5.8 kb in *Diphylleia rotans*, here named Diphy I (calculated from genome sequence)*,* ~5.9 kb in Å85 and KIVT03, here named Diphy II, to ~6.3 kb in KIINB, KIKNR01, KIKNR02, KIKNR03, KIVT01, KIVT02, KIVT04, KIVTT01 and KIVTT02, here named Diphy III. As such, the three groups (Diphy I, II and III) had a 69–79% pairwise identity over the amplicon length, and a 73–86% pairwise identity for the 18S rRNA gene.

### Diphyllatea diversity in natural samples

To investigate the diversity of the class further we designed new Diphyllatea-specific primer pairs and applied these to environmental DNA samples from various habitats. The presence of 18S rRNA was confirmed in all environmental samples using the universal eukaryote primers NSF83 - 1528R. The Diphyllatea-specific primer pair Diphy257F - Diphy1881R, designed in this study, successfully amplified 18S rRNA from four of eight environmental freshwater samples (Table 3). The primer pair with lower specificity to known Diphyllatea 18S rRNA, Diphy453F – 1528R, successfully amplified template from a further three of the freshwater samples (Table 3).

**Fig. 3:**
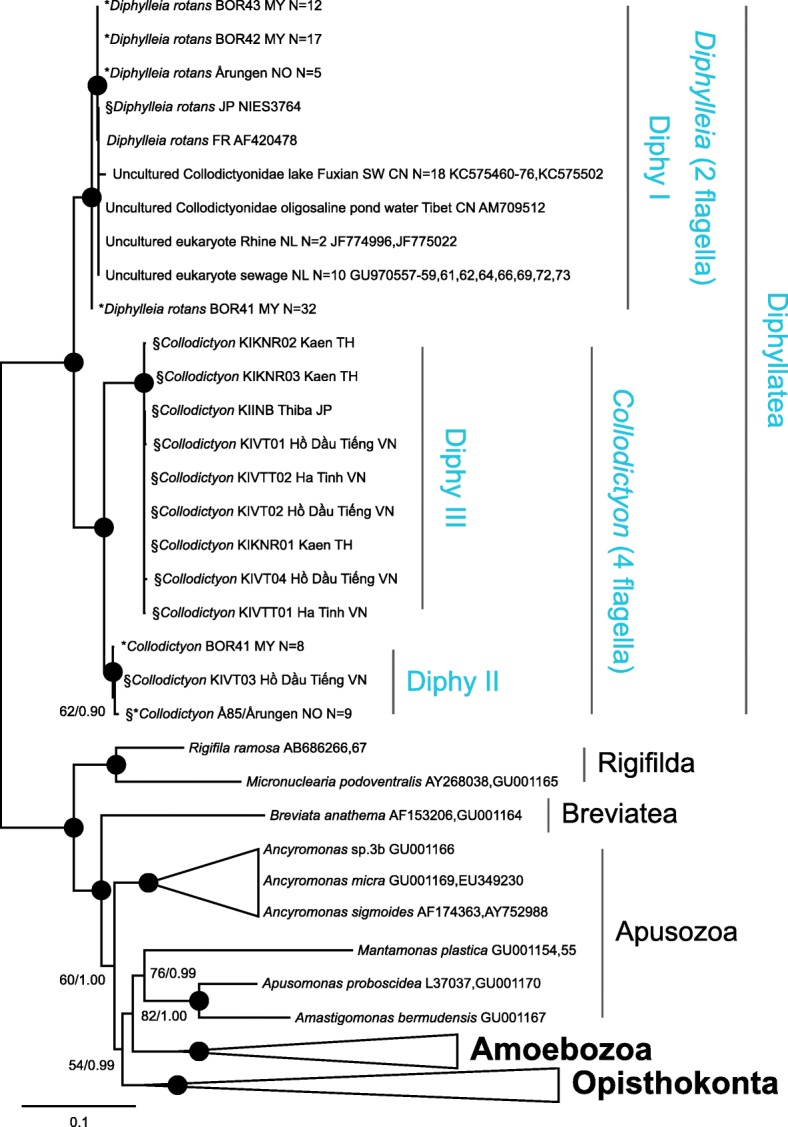
The rRNA phylogeny of Diphyllatea. The topology was reconstructed with the GAMMA-GTR model in RAxML v8.0.26. and inferred with 64 taxa and 3983 characters. The inference has been collapsed at varying taxonomic levels for easier visualisation, with blue representing the in-group. The numbers on the internal nodes are ML bootstrap values (BP, inferred by RAxML v8.0.26. under then GAMMA-GTR model) and posterior probabilities (PP, inferred by MrBayes v3.2.2 under the GTR + GAMMA+Covarion model), ordered; RAxML/MrBayes. Black circles indicate BP > 90% and PP 1.00, values with BP < 50% are not shown. Asterisk (*) denotes environmental OTUs sequenced in this study, with “N” representing the number of reads included in each OTU. § depicts rRNA from cultured Diphyllatea amplified in this study. The clonal Å85 Sanger sequence and Årungen PacBio OTU are represented as a single taxon as they shared a 100% identity. Abbreviations for countries: CN = China, FR = France, JP = Japan, MY = Malaysia, NL = Netherlands, NO = Norway, TH = Thailand, and VN = Vietnam. See Additional file 1: Figure S2 for 18S rRNA inference of Diphyllatea

Sequencing these environmental amplicons on a SMRTcell confirmed Diphy257F - Diphy1881R successfully amplified targeted 18s rRNA from Diphyllatea, whilst Diphy453F – 1528R was unsuccessful in amplifying Diphyllatea template (Table 3). Pacbio CCS reads were filtered and clustered at a 98% identity threshold before being aligned with the Diphy I-III 18s – 28s rRNA fragments previously amplified (see alignment and Fig. 3). The freshwater samples BOR41 and Årungen both showed the presence of Diphy I and II sequences. It should be noted that the Årungen Diphy II PacBio OTU and the clonal Å85 Sanger sequence shared a 100% identity over sequence length. Only Diphy I sequence data was observed in the BOR42 and BOR43 samples, though this is a likely result of limited sequencing depth. None of our new environmental 18S rRNA gene amplicons (MF039351–55) showed similarity to the Diphy III sequence. No additional sequences were obtained that clustered external to the three Diphyllatea groupings already confirmed (Diphy I-III). Only a single Diphyllatea OTU for each clade was recovered from the individual environmental samples using a 98% clustering identity threshold (Fig. 3). A such a more relaxed threshold would not have affected the recovered diversity.

The new primers Diphy257F and Diphy1881R therefore provide a promising tool for future investigations of the Diphyllatea diversity. It should be noted, however, that even though Diphyllatea 18S rRNA was successfully amplified, the new primers also amplified a putative protein-coding gene (4-diphospocytidyl-2C-methyl-D-erythritol kinase) from a possible novel brackish Actinobacteria (MF039368) in two samples (BOR41 and BOR43). As these sequences were not 18S rRNA, and obviously of prokaryote origin, they were discarded.

### Diphyllatea unconfirmed in marine habitats

To date, Diphyllatea species have only been observed in freshwater environments, including an estuary of a freshwater river [13]. As the new PCR primers successfully amplify Diphyllatea 18S rRNA from environmental samples, we used them to investigation a possible cryptic diversity in marine environments. However, while the marine DNA samples were of good quality and previously used for large surveys of protist diversity [53], we were unable to amplify Diphyllatea 18S rRNA (Table 3) using the Diphy257F - Diphy1881R primer pair. Applying less stringent PCR conditions (i.e. lowering annealing temperature from 55 °C to 50 °C) had no effect on this result. As previous, and in an attempt to amplify novel Diphyllatea template rRNA with lower sequence identity, the primer pair Diphy453F - 1528R was used, successfully amplifying product from three of the four marine DNA samples (Table 3). However, once these environmental amplicons had been sequenced on a SMRTcell, filtered, and clustered, none of the 192 OTUs they represented had an affinity to Diphyllatea (see Table 3 and Additional file 1: Figures S2 and S4).

In our search for marine Diphyllatea sequences, we complemented our PCR and sequencing approach by searching public sequence databases. Despite querying four of the largest sequence databases (BioMarKs, GOS, Tara oceans marine metagenome, and Tara oceans V9) for the presence of Diphyllatea rRNA and two databases (GOS and Tara oceans marine metagenome) with 124 *Collodictyon* gene transcripts [1], we were unable to identify any marine Diphyllatea-like sequences. Instead, we identified 30 freshwater 18S rRNA sequences in the NCBInr database additional to the known *Diphylleia rotans* sequence from France (AF420478) and the uncultured Collodictyonidae sequence from Tibet (AM709512): 18 sequences (KC575460–76, KC575502) were from Lake Fuxian SW China, 2 sequences (JF774996, JF775022) from Rhine river water, Netherlands, and 10 sequences (GU970557–59, 61, 62, 64, 66, 69, 72, 73) from sewage water, Netherlands. All showed highest pairwise affinity to Diphy I (Fig. 3).

### Phylogeny and diversity of the class Diphyllatea

Using all generated and acquired sequences in phylogenetic reconstruction (Fig. 3) resulted in a monophyletic Diphyllatea grouping with full bootstrap support (BS) and posterior probability (PP). Further, Diphyllatea formed a fully supported (100 BS / 1.00 PP) clade with *Rigifila ramosa* and *Micronuclearia podoventralis* (order Rigifilda) [5, 9].

Sequences within Diphyllatea (Fig. 3) were divided into three clades, all fully supported. Two of these include the previously known genera *Diphylleia* and *Collodictyon*, here marked Diphy I and II respectively. Furthermore, and most importantly, the tree reveals a new Diphyllatea clade distinct from the previous two. The new clade, here named Diphy III, branches off as a sister clade to Diphy II with full support, though both clades represent species with a comparable *Collodictyon* morphology. All 11 cultured strains are placed within *Collodictyon* (Diphy II and III); two placing within Diphy II (Å85 and KIVT03), and the remaining nine within Diphy III (KIINB, KIKNR01, KIKNR02, KIKNR03, KIVT01, KIVT02, KIVT04, KIVTT01 and KIVTT02).

To increase phylogenetic resolution of the ingroup, the phylogeny was additionally inferred without outgroup taxa (Additional file 1: Figure S3). This inference was equivalent to that of Fig. 1, with higher support for the observed branching patterns. The major difference between the two analyses was the separation of Diphy III strains into two supported clades.

## Discussion

### Cultured Diphyllatea show a *Collodictyon* morphology

Diphyllatea, dependent on the position of the eukaryotic root, may represent one of the earliest branching eukaryote lineages, and as such is of pivotal importance for reconstructing eukaryotic evolution. Still, virtually nothing is known about the higher order phylogenetic structure, diversity and geographic dispersal of this deep lineage. Here we address these issues by combining culturing, rRNA sequencing of new strains and targeted 18S rRNA PCR of natural samples sequenced with PacBio RS II technology.

Morphologically these isolates all seem to be strains of *C. triciliatum* or closely related species, supporting earlier studies that Diphyllatea encompasses a limited species number [8, 11–13]. Alternatively, a similar phenotype may represent multiple cryptic species with distinct genotypes (or ribotypes).

### Diphyllatea unconfirmed in marine habitats

Altogether, our PCR amplification and database searches could only detect Diphyllatea in freshwater, suggesting that the class maybe restricted to freshwater habitats. Knowledge of Diphyllatea habitat preferences and distributions in environmental systems remains limited. Therefore, the development of targeted PCR approaches, presented here, can be useful in future studies on additional environments.

### Phylogeny and diversity of the class Diphyllatea

As we show that the *C. triciliatum* morphospecies concept encompasses two distinct molecular clades within Diphyllatea, future microscopy work should establish what taxonomic levels these represent, and if possible, which is most congruent with the morphological description of the type species. The taxonomic rank Diphy I-III is not clear, but all groups contain higher diversity than earlier known, and several substructures that might constitute different sub-groups It should also be noted that Diphy III is likely to be a clade at least on the same taxonomic level as Diphy I and II. Considering the sequence divergence between Diphy II and III: Only 79% sequence identity was shared over the rRNA length, increasing to 85% for the more conserved 18S rRNA region, suggesting these clades are at least separate genera with a shared morphology. Another distinct pattern in the tree, is the placement of *Diphylleia* BOR41 environmental OTU, which placed as sister to Diphy I and excluded from this clade with almost full support (99/1.00), and 95 BS in Additional file 1: Figure S3, but still showed > 98% pairwise identity to the other OTUs in Diphy I, suggesting *Diphylleia* likely constitutes several uncharacterized cryptic species (Fig. 3 and Additional file 1: Figure S3).

Mapping the morphology to the tree shows that the quadriflagellate forms branch together as two main monophyletic groups (i.e. Diphy II and III), implying that Diphyllatea as a group is deeply divided into two clades composed of quadriflagellate or biflagellate (Diphy I) forms. As the flagella of *Collodictyon* occupy the same position as the basal body in a pre-division stage of *Diphylleia* [6], and the cyst stage has two long gelatinous filaments [64], it could be hypothesised that the two forms represent different life-stages of the same species. However, no biflagellate stage was observed for our Diphy II and III cultures, with the phylogeny consistently separating the biflagellate *Diphylleia* from the two clades of quadriflagellate *Collodictyon* species. Hence, the morphological change in Diphyllatea has most likely occurred early in the history of the group. Dependent on the final position of the eukaryote root, this event may represent one of the most ancient morphological innovations known among eukaryotes.

Currently, available sequence data suggests a single biflagellate clade (Diphy I). However, additional sequence data is needed to confirm that *Sulcomonas lacustris* and possible cryptic Diphyllatea species place within the Diphy I clade.

To further understand the evolution of Diphyllatea, the ancestral form and its relationship within Sulcozoa, the genomes of multiple species are essential. For this reasoning, we are currently completing the annotation of genomes from each of the three Diphyllatea clades, in addition to that of *Rigifila ramosa* (Rigifilda).

### A global distribution of Diphyllatea

The substantial increase in Diphyllatea sequence data presented, allows, for the first time, conclusions to be drawn as to the extent and ecological role of the class and genera therein.

The Diphy I clade (Fig. 3), representing the biflagellate cell-type, constituted sequences from Borneo, China, France, Japan, Netherlands and Norway, including the previously reported ribotypes from Tibet (China) and Clermont-Ferrand (France) [6, 18]. In addition to the localities above, *Diphylleia* has also be reported in Saudi Arabia [15], suggesting a global distribution of the genus.

The Diphy II clade constituted sequences from Borneo and Norway, from both culture and environmental samples. The Diphy III clade, in contrast, constituted only cultured sequence data from Asia (Japan, Thailand and Vietnam) with no environmental Diphyllatea amplicons having an affinity to this clade. *Collodictyon*, the quadriflagellate form (represented in Diphy II and III clades; Fig. 3), has been previously described from the island of Bombay and later in central Europe, Spain and Norway [11, 14, 23, 24]. The quadriflagellate morphotype has additionally been reported in North America [11, 22, 23], and more recently South America; from multiple freshwater localities in Uruguay: La Oriental, Maldonado (34°34’S 55°15’W), Tala, Canelones (34°20’S 55°45’W), and Picada Varela, San José River, San José (34°19’S 56°42’W). Accompanying video is available through https://www.youtube.com/ (uuvb3eUZUQ8, AsY8s-HnTMQ, M8tAf3KoDQM and k88LsRcEXmg). As only morphological data is presented, in the reports above, we are unable to establish if these morphotypes represent Diphy II and/or III, however it does confirm a global distribution of the *Collodictyon* morphotype and accordingly the Diphyllatea class.

Interestingly, all environmental sequence data for Diphyllatea deposited in public databases were only related to the Diphy I clade. The reason for this pattern is unlikely a PCR primer bias, as the used primers show full match to all three Diphy groups. It may rather indicate higher abundance of Diphy I in the sampled localities or could reflect different habitat preferences among the three Diphy groups.

### PacBio SMRT sequencing of targeted Diphyllatea amplicons

The PacBio RS sequencing platform has been previously used to study 16S [39, 40] and more recently 18S rRNA gene amplicons [41, 42]. Jones and Kustka sequenced the V7–9 region of 18S rRNA to answer questions about total eukaryotic diversity from marine samples [41]. Tedersoo et al., targeted the V4 (18S) - D3 (28S) region focusing on total eukaryotic and fungal diversity from soil samples, confirming PacBio as an alternative for metabarcoding of organisms with low diversity for reliable identification and phylogenetic approaches [42]. In contrast to these studies, which surveyed a broader eukaryotic diversity, our goal was a targeted 18S rRNA approach. Our result demonstrates PacBio RS as an efficient and economical alternative to the traditional cloning and Sanger method for sequencing long rRNA amplicons [32–34]; A single SMRT cell gave 6310 total reads of insert (Additional file 1: Table S2), which constituted 1741 high quality sequences (CCS = 1) and 281 OTUs. To achieve a comparable number of sequences via cloning and Sanger sequencing would be a tedious exercise, at an approximate cost 30× higher than that of the PacBio RS method (based on CCS = 1 result).

The major advantage of PacBio RS for the study eukaryotic of diversity is read length, which allows for higher phylogenetic resolution. Additionally, long-reads allow short-read amplicon datasets from different rRNA regions to be “scaffolded” and inferred in parallel, further increasing resolution. However, PacBio RS does have a high error rate, ~15% with the P4-C2 chemistry due to the random addition of incorrect nucleotides [65]. To overcome the random error rate, DNA is ligated into SMRT bells; circular DNA fragments that allow multiple sequence passes, a process termed Circular Consensus Sequencing (CCS), and further accounted for with a sequence analysis pipeline. Our results demonstrate an analysis pipeline as essential for the removal of sequencing errors that can affect variability and show the Årungen PacBio OTU and the clonal Å85 Sanger sequence to be 100% identical over sequence length. Additionally, chimeras, that increase proportionally with amplicon length [42, 66] can give an overestimation of diversity; Of the 1741 high quality sequences, 186 (11.44%) were identified as chimeric using Uchime (Additional file 1: Table S2). The high level of chimeric sequences identified was surprising, with only a 1–2% chimera level previously reported [39, 65] by “misligation” of SMRTbell adaptors in the PacBio library preparation. The observed chimera level is therefore attributed to PCR artefacts; It has been proposed that > 45% of reads in some datasets are chimeric [66–68], with experiments showing that > 30% of chimeras can be attributed to PCR [69]. Formation of PCR chimeras increases with amplicon length, a result of template switching [66], as such filters to identify and remove long-read amplicon chimeras (i.e. Uchime) are paramount. It is difficult to ascertain if the observed chimeras are a result of the original 18S rRNA amplification or the subsequent PCR to attach symmetric PacBio barcodes. It is possible, however to reduce the former by decreasing amplification cycles [70], and eradicate the latter by barcode ligation or the sequencing of separate samples, instead of multiplexing. It has been previously reported that Uchime fails to identify all long-read amplicon chimeras [39], though we found no evidence supporting this in our dataset.

In agreement with previous studies [41, 42], we find that long-read PacBio RS sequencing provides high phylogenetic resolution for eukaryotic diversity studies. Further, PacBio will improve with technological and chemical developments, allowing for longer and more accurate reads with higher output [39], with the recent release of PacBio Sequel confirming [42, 71]. Understanding PacBio biases will allow for improved bioinformatic pipelines, and as such phylogenetic inferences [39]. It should be noted that Oxford Nanopore sequencing platform has recently been applied to 16S rRNA gene amplicons [72], and may offer an alternative for the study of eukaryotic diversity with long-reads.

## Conclusions

In this study, the application of culturing Diphyllatea protists in parallel with Sanger sequencing the partial rRNA operon from multiple strains, reveals two quadriflagellate ribotypes despite only a single morphotype being observed. Further, the inference of Diphyllatea cultured sequences with that of database orthologues and environmental amplicons infers a greater Diphyllatea diversity than previously known, recovering three clearly phylogenetically separated groupings (Diphy I, II, and III). The substantial addition of sequence data, in this study, resolves relationships between genera. We show a split between the Diphy I and Diphy II + III clades corresponding to a morphological division of Diphyllatea into bi- and quadriflagellate cell forms. The altered Diphyllatea morphology most likely occurred early in the history of the group and may represent one of the most ancient morphological innovations known among eukaryotes. Furthermore, environmental sequences and database mining show a global distribution of Diphyllatea with a dispersal restricted to freshwater habitats.

Our results suggest that combining culture methods with a group-specific PCR approach and long-read sequencing is invaluable for understanding the diversity and distribution of protist lineages, in particular Diphyllatea, and their ecological importance in aquatic systems. Here we provide the tools to uncover the true diversity of this class.

Lastly, our study shows the capacity of PacBio RS when employing a targeted approach for increasing phylogenetic resolution of a protist class. Although caution needs to be observed when analysing reads, to avoid a chimeric overestimation of diversity, the platform offers major economical and efficiency gains over traditional cloning and Sanger sequencing methods, something that will improve with technological advances.

## Additional files

Additional file 1: **Table S1.** Sanger sequencing primers. **Table S2.** Sequencing results for environmental amplicons per barcode. **Figure S1.** PacBio sequencing results. **Figure S2.** The 18S rRNA phylogeny of Diphyllatea. **Figure S3.** The rRNA phylogeny of Diphyllatea excluding outgroup taxa. **Figure S4.** Total diversity of generated OTUs. (DOCX 1098 kb)

Additional file 2: **Video S1**. Motile *Collodictyon* cell. With relaxed movement and rotation driven by flagella. *Collodictyon* Å85 strain is shown. Video is filmed using a Nikon D300S on a Nikon Diaphot inverted microscope. (M4V 3804 kb)

Additional file 3: **Video S2**. Cytoplasmic veil and pseudopodia. A *Collodictyon* cell clinging to the surface of the culture dish by a cytoplasmic veil and pseudopodia (i.e. the amoeboid property). *Collodictyon* Å85 strain is shown. Video is filmed using a Nikon D300S on a Nikon Diaphot inverted microscope. (M4V 6311 kb)

## Abbreviations

BS: Bootstrap support
CCS: Circular consensus sequencing
DIC: Differential interference contrast
EM: Electron microscopy
GOS: Global ocean sampling
MCMC: Markov Chain Monte Carlo
NHM: Natural History Museum, London
NIVA: Norwegian Institute for Water research
NSC: Norwegian Sequencing Centre
OTU: Operational taxonomic unit
PacBio: Pacific Bioscience
PCR: Polymerase Chain Reaction
PP: Posterior probability
UoO: University of Oslo
