# Correction to: Enigmatic Diphyllatea eukaryotes: culturing and targeted PacBio RS amplicon sequencing reveals a higher order taxonomic diversity and global distribution

**DOI:** 10.1186/s12862-018-1233-y

**Published:** 2018-08-03

**Authors:** Russell J. S. Orr, Sen Zhao, Dag Klaveness, Akinori Yabuki, Keiji Ikeda, Makoto M. Watanabe, Kamran Shalchian-Tabrizi

**Affiliations:** 1 Section for Genetics and Evolutionary Biology (EVOGENE), Department of Biosciences, University of Oslo, Kristine Bonnevies hus, Blindernveien 31, 0371 Oslo, Norway; 2 Centre for Integrative Microbial Evolution (CIME), Section for Genetics and Evolutionary Biology (EVOGENE), Department of Biosciences, University of Oslo, Kristine Bonnevies hus, Blindernveien 31, 0371 Oslo, Norway; 3 Department of Molecular Oncology, Institute of Cancer Research, Oslo University Hospital-Radiumhospitalet, Oslo, Norway; 4 Medical Faculty, Center for Cancer Biomedicine, University of Oslo University Hospital, Oslo, Norway; 5 Section for Aquatic Biology and Toxicology (AQUA), Department of Biosciences, University of Oslo, Oslo, Norway; 6 Japan Agency for Marine-Earth Science and Technology (JAMSTEC), 2-15 Natsushima, Yokosuka, Kanagawa 237-0061 Japan; 7 Faculty of Life and Environmental Sciences, University of Tsukuba, 1-1-1 Tennodai, Tsukuba, Ibaraki 305-8572 Japan

## Correction

In the original publication of this article [1] there was an error in an author name. In this correction article the correct and incorrect name are indicated.

Incorrect name upon publication:

- Watanabe M. Makoto

Corrected name:

- Makoto M. Watanabe

The original publication has been updated. The publisher apologizes to the readers and authors for the inconvenience.
