# Supplementary material for: Enigmatic Diphyllatea eukaryotes: culturing and targeted PacBio RS amplicon sequencing reveals a higher order taxonomic diversity and global distribution
Source: BMC Evol Biol. 2018 Jul 18;18:115. doi: 10.1186/s12862-018-1224-z (PMC6052632; doi:10.1186/s12862-018-1224-z)
Supplement: Supplementary file 1 — Table S1. Sanger sequencing primers. Table S2. Sequencing results for environmental amplicons per barcode. Figure S1. PacBio sequencing results. Figure S2. The 18S rRNA phylogeny of Diphyllatea. Figure S3. The rRNA phylogeny of Diphyllatea excluding outgroup taxa. Figure S4. Total diversity of generated OTUs. (DOCX 1098 kb) [file 12862_2018_1224_MOESM1_ESM.docx]

**Additional files**

| **Primer name** | **Primer direction** | **Primer sequence (5’-3’)** | ***Tm* (^o^C)** | **Annealing site (5’-3’)** | **Reference or source** |
| --- | --- | --- | --- | --- | --- |
| NSF83 | F | GAAACTGCGAATGGCTCATT | 49.7 | 84-103 | [1] |
| 528F | F | CGGTAATTCCAGCTC | 41.9 | 595-609 | [2] |
| 18s-EUK-1134-F | F | CGCAAGGCTGAAACTTAAA | 46.8 | 1324-1342 | Adapted from [3] |
| NSF1624 | F | TTTGYACACACCGCCCGTCG | 55.9 | 1973-1992 | [4] |
| Euk_B_F | F | AGGTGAACCTGCAGAAGGATCA | 54.8 | 2129-2150 | Adapted from [5] |
| SR1 | R | CGGTACTTGTTCGCTATC | 48 | 3565-3583 | Ema Chao pers. comm |
| 28S_m_F | F | TGGGACCCGAAAGACAGTGA | 53.8 | 4084-4103 | Modified from Ando et al (2009) |
| TW14R | R | GCTATCCTGAGGGAAACTTC | 51.8 | 4237-4256 | [6] |
| 28S_4803F | F | CAAGTGAGATCCTTGAAGACTG | 53.0 | 4803-4824 | This study |
| 28S_5761F | F | ACGGCGGGAGTAACTATGAC | 53.8 | 5761-5781 | This study |
| 28S_5781R | R | GTCATAGTTACTCCCGCCGT | 53.8 | 5761-5781 | This study |
| LR11 | R | GCCAGTTATCCCTGTGGTAA | 51.8 | 6414-6433 | [7] |

**Table S1. Sanger sequencing primers:** Primer annealing site is based on *Collodictyon* KIVT02 sequence, start is 83bp prior to account for NSF83s annealing site. *Tm* is calculated using oligocalc. Amplification primers are listed in Table 2.

| **Barcode number** | **Sample** | **Total reads of insert** | **Filtered reads** | **Unique filtered reads** | **Chimeras** | **OTUs** | **OTUs >1** |
| --- | --- | --- | --- | --- | --- | --- | --- |
| 7 | BOR41 (Diphy257F-1881R), NB038 (Diphy453F-1528R) | 2908 | 747 | 706 | 99 | 158 | 14 |
| 10 | BOR42 (Diphy257F-1881R), RA119 (Diphy453F-1528R) | 1277 | 319 | 315 | 66 | 58 | 9 |
| 17 | + control (Diphy453F-1528R) | 76 | 20 | 20 | 1 | 4 | 3 |
| 19 | BOR43 (Diphy257F-1881R), 20F268 (Diphy453F-1528R) | 1719 | 596 | 526 | 16 | 32 | 8 |
| 32 | SA78, 81 (Diphy453F-1528R) | 56 | 9 | 9 | 0 | 7 | 1 |
| 39 | Årungen (Diphy257F-1881R) LD_BASS2, 20 (Diphy453F-1528R) | 202 | 34 | 34 | 4 | 14 | 4 |
| 45 | LD_ESTH20 (Diphy453F-1528R) | 72 | 16 | 16 | 0 | 8 | 2 |
| **TOTAL** | **11** | **6310** | **1741** | **1626** | **186** | **281** | **41** |

**Table S2.** **Sequencing results for environmental amplicons per barcode:** Filtered reads are those with a CCS quality of 1. Chimeras are calculated from the unique filtered reads. The PacBio barcodes used in this study are listed here: <https://github.com/PacificBiosciences/Bioinformatics-Training/blob/master/barcoding/pacbio_barcodes_paired.fasta>. A total of seven barcodes were used in this study, thus multiple amplicons were sequenced with the same barcode. To allow for sample separation, identical barcodes were only used for amplicons from different primer-pairs.

**Supporting Figures**

**Figure S1.** **PacBio sequencing results.** The sequencing results for the single sequenced SMRTcell (PacBio RS II P4-C2 chemistry) showing from left to right; Read Length of Insert, Read Quality of Insert, and Number of Passes.


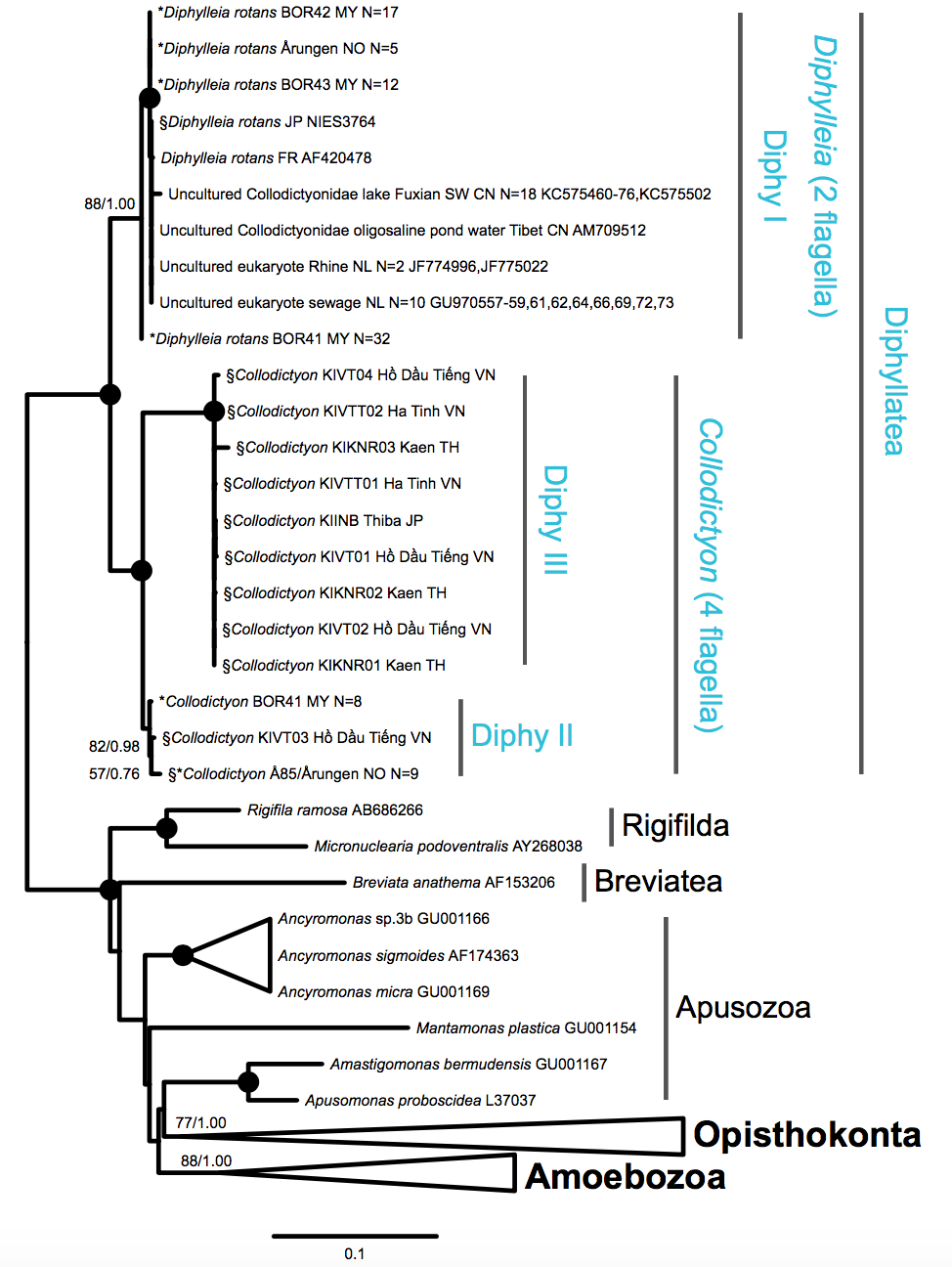


**Figure S2.** **The 18S rRNA phylogeny of Diphyllatea.** The topology was reconstructed with the GAMMA-GTR model in RAxML v8.0.26. and inferred with 64 taxa and 1,575 characters. The inference has been collapsed at varying taxonomic levels for easier visualisation, with blue representing the in-group. The numbers on the internal nodes are ML bootstrap values (BP, inferred by RAxML v8.0.26. under then GAMMA-GTR model) and posterior probabilities (PP, inferred by MrBayes v3.2.2 under the GTR+GAMMA+Covarion model), ordered; RAxML/MrBayes. Black circles indicate BP > 90% and PP 1.00, values with BP < 50% are not shown. Asterisk (*) denotes environmental OTUs sequenced in this study, with “N” representing the number of reads included in each OTU. § depicts rRNA from cultured Diphyllatea amplified in this study. The clonal Å85 Sanger sequence and Årungen PacBio OTU are represented as a single taxon as they shared a 100% identity. Abbreviations for countries: CN = China, FR = France, JP = Japan, MY = Malaysia, NL = Netherlands, NO = Norway, TH = Thailand, and VN = Vietnam. See Fig. 3 for rRNA inference of Diphyllatea.

**
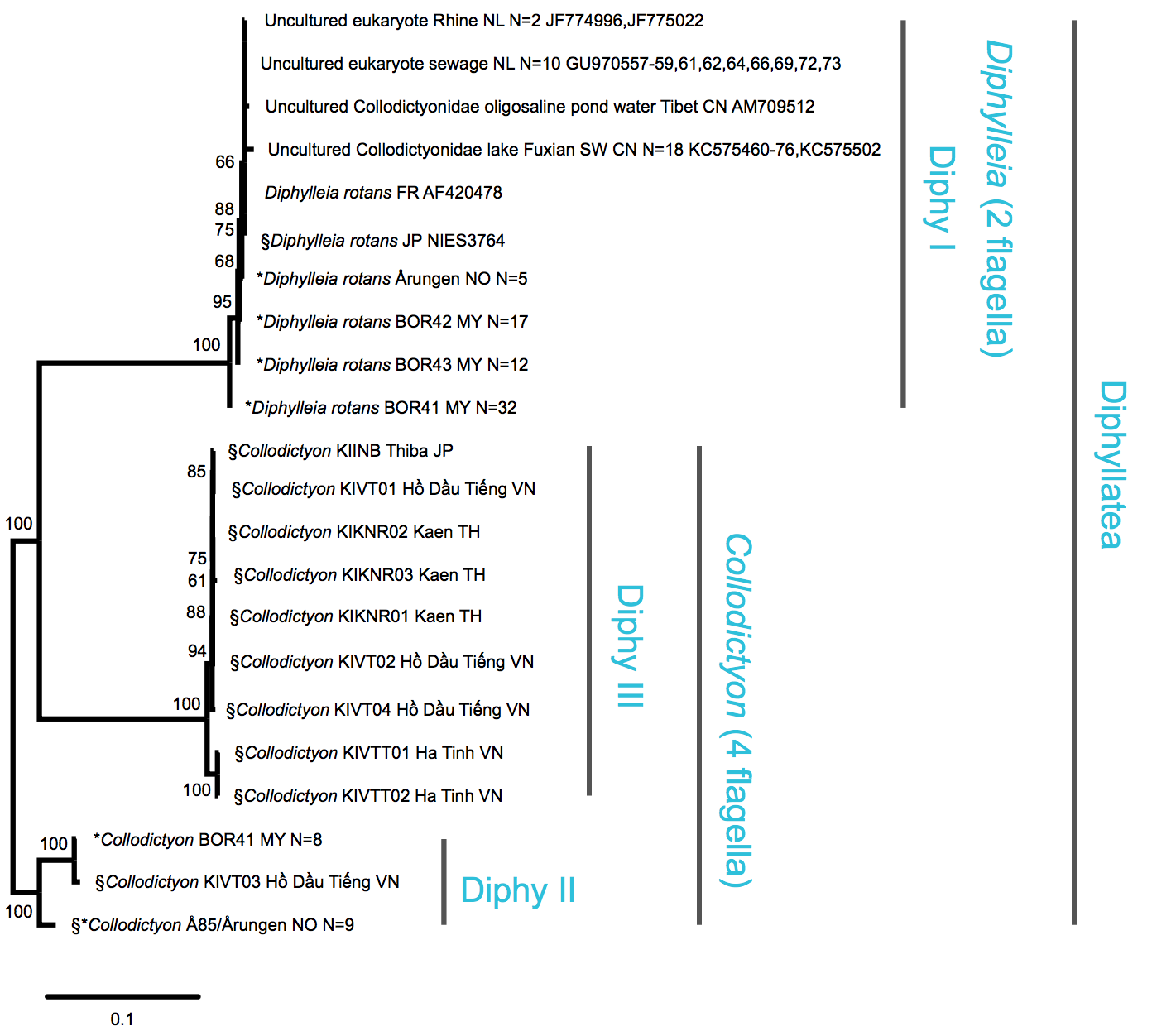
**

**Figure S3.** **The rRNA phylogeny of Diphyllatea excluding outgroup taxa.** The topology was reconstructed with the GAMMA-GTR model in RAxML v8.0.26. and inferred with 22 ingroup taxa and 6,795 characters. The inference has been collapsed at varying taxonomic levels for easier visualisation. The numbers on the internal nodes are ML bootstrap values (BP, inferred by RAxML v8.0.26. under then GAMMA-GTR model). Asterisk (*) denotes environmental OTUs sequenced in this study, with “N” representing the number of reads included in each OTU. § depicts rRNA from cultured Diphyllatea amplified in this study. The clonal Å85 Sanger sequence and Årungen PacBio OTU are represented as a single taxon as they shared a 100% identity. Abbreviations for countries: CN = China, FR = France, JP = Japan, MY = Malaysia, NL = Netherlands, NO = Norway, TH = Thailand, and VN = Vietnam. See Supplementary Fig. 2 for 18S rRNA inference of Diphyllatea.


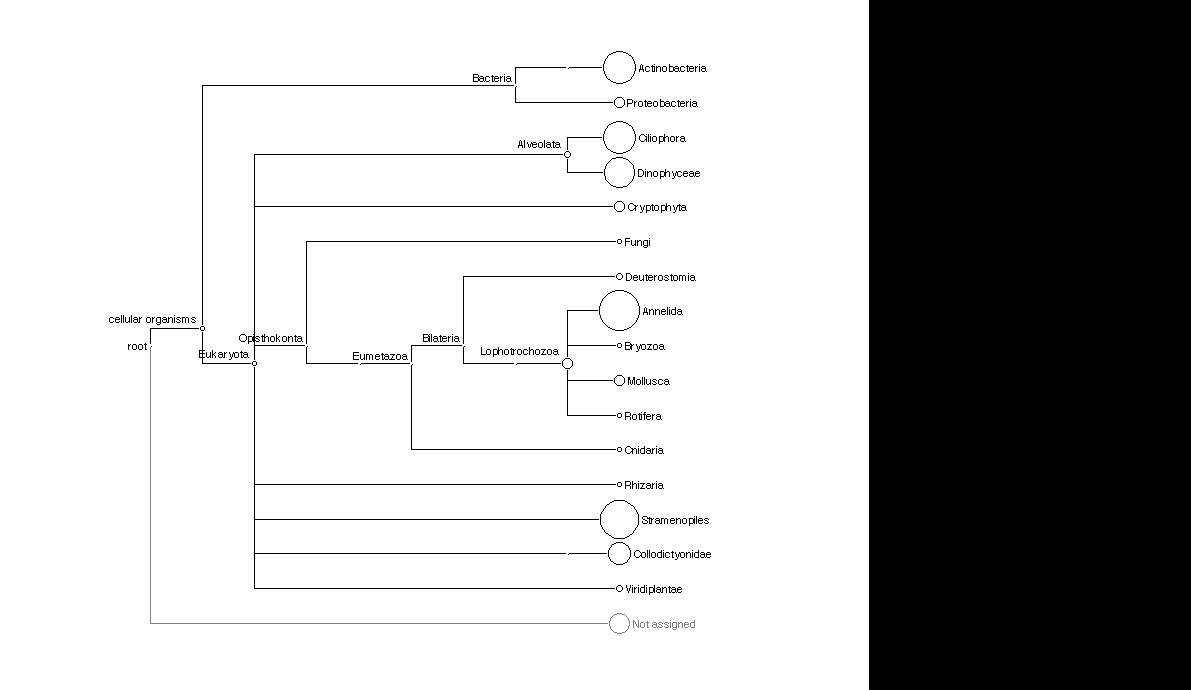

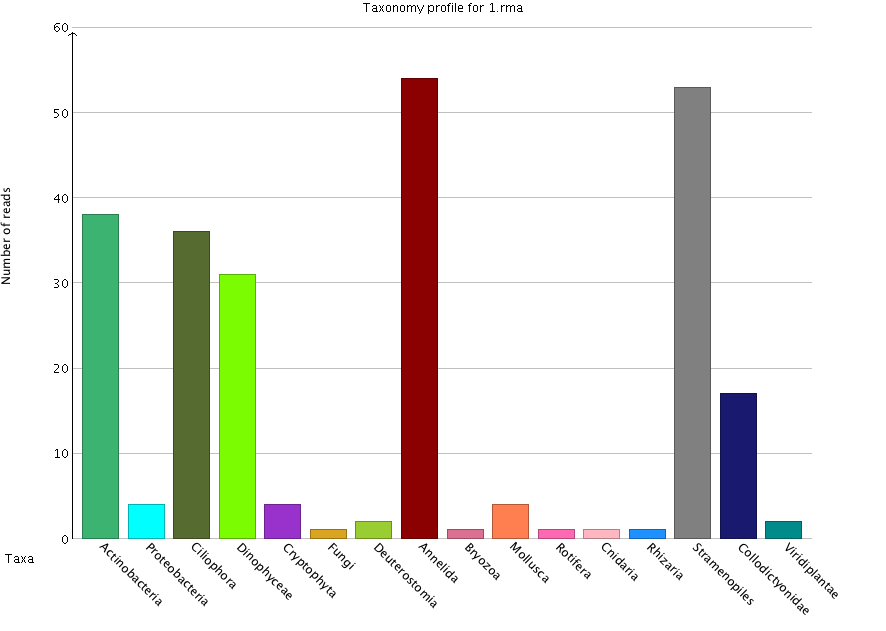


**Figure S4.** **Total diversity of generated OTUs.** The blastn result for all 281 OTUs generated from this study is represented on the left as a cladogram at different taxonomical ranks. The circle size is proportional to the number of OTUs that have been assigned to each respective taxonomical rank. On the right the result is presented as a bar-chart with the X-axis representing the same taxonomical ranks as the cladogram and the Y-axis representing the number of OTUs assigned to each. Blastn was performed against the NCBInr database with default parameters (15.4.18) using CLC main workbench 7 (Qiagen). The cladogram and bar-chart showing the total diversity of generated OTUs was produced using MEGAN [8]. “Collodictyonidae” refers to Diphyllatea OTUs. A fasta file containing all 281 OTUs is provided through the authors’ ResearchGate page: <https://www.researchgate.net/home>

**REFERENCES:**

1. Hendriks L, Goris A, Neefs JM, Van de Peer Y, Hennebert G, Dewachter R: **The Nucleotide-Sequence of the Small Ribosomal-Subunit RNA of the Yeast *Candida albicans* and the Evolutionary Position of the Fungi among the Eukaryotes**. *Systematic and Applied Microbiology* 1989, **12**(3):223-229.

2. Edgcomb VP, Kysela DT, Teske A, de Vera Gomez A, Sogin ML: **Benthic eukaryotic diversity in the Guaymas Basin hydrothermal vent environment**. *Proc Natl Acad Sci U S A* 2002, **99**:7658-7662.

3. Bower SM, Carnegie RB, Goh B, Jones SRM, Lowe GJ, Mak MWS: **Preferential PCR Amplification of Parasitic Protistan Small Subunit rDNA from Metazoan Tissues**. *Journal of Eukaryotic Microbiology* 2004, **51**(3):325-332.

4. Van der Auwera G, Chapelle S, De Wächter R: **Structure of the large ribosomal subunit RNA of Phytophthora megasperma, and phylogeny of the oomycetes**. *FEBS Letters* 1994, **338**(2):133-136.

5. Medlin L, Elwood HJ, Stickel S, Sogin ML: **The Characterization of Enzymatically Amplified Eukaryotic 16s-Like Rrna-Coding Regions**. *Gene* 1988, **71**(2):491-499.

6. Cullings K: **Molecular phylogeny of the Monotropoideae (Ericaceae) with a note on the placement of the Pyroloideae**. *Journal of Evolutionary Biology* 1994, **7**(4):501-516.

7. Schmitt I, Crespo A, Divakar PK, Fankhauser JD, Herman-Sackett E, Kalb K, Nelsen MP, Nelson NA, Rivas-Plata E, Shimp AD *et al*: **New primers for promising single-copy genes in fungal phylogenetics and systematics**. *Persoonia* 2009, **23**:35-40.

8. Huson DH, Mitra S, Ruscheweyh H-J, Weber N, Schuster SC: **Integrative analysis of environmental sequences using MEGAN4**. *Genome Research* 2011, **21**(9):1552-1560.
